# HIV Integration into the PTEN Gene and Its Tumor Microenvironment Implications for Lung Cancer

**DOI:** 10.3390/curroncol32070389

**Published:** 2025-07-04

**Authors:** Davey M. Smith, Elizabeth F. Rowland, Sara Gianella, Sandip Pravin Patel, Stephanie Solso, Cheryl Dullano, Robert Deiss, Daria Wells, Caroline Ignacio, Gemma Caballero, Magali Porrachia, Collin Kieffer, Antoine Chaillon

**Affiliations:** 1 Department of Medicine, University of California, San Diego, CA 92093, USA; d13smith@health.ucsd.edu (D.M.S.); gianella@health.ucsd.edu (S.G.); spatel@health.ucsd.edu (S.P.P.); ssolso@health.ucsd.edu (S.S.); cdullano@health.ucsd.edu (C.D.); rgdeiss@health.ucsd.edu (R.D.); cignacio@health.ucsd.edu (C.I.); g1caballero@health.ucsd.edu (G.C.); mporrachia@health.ucsd.edu (M.P.); 2 Department of Microbiology, University of Illinois at Urbana-Champaign, Urbana, IL 61820, USA; efrowla2@illinois.edu; 3 Leidos Biomedical Research, Inc., Frederick National Laboratory for Cancer Research, Frederick, MD 21702, USA; wellsd1984@gmail.com; 4 Department of Bioengineering, University of Illinois at Urbana-Champaign, Urbana, IL 61820, USA

**Keywords:** HIV, lung cancer, integration, tumor microenvironment, clonal expansion, PTEN

## Abstract

Health outcomes for people with HIV (PWH) have improved significantly with combination antiretroviral therapy (ART), yet the risk of lung cancer remains elevated. While a single case cannot establish causality, we describe here an investigation of a 74-year-old male PWH with de novo high-grade neuroendocrine small cell lung carcinoma. To investigate the potential contribution of HIV to cancer development, we performed HIV integration site sequencing on blood, tumor, and non-tumor tissue samples from the patient. We analyzed integration site distribution, clonal expansion, and associated gene disruption. Phosphatase and Tensin Homolog (PTEN) expression was evaluated using immunofluorescence and microscopy. A total of 174 unique HIV integration sites were identified, with 29.9% (52/174) located in clonally expanded cells. The most frequent integration site in clonally expanded cells was within the PTEN gene, representing 4.2% to 16.7% of all HIV-infected cells across samples. PTEN expression was markedly reduced in tumor regions relative to non-tumor tissue. Areas positive for HIV p24 antigen showed minimal PTEN expression. These findings suggest that HIV integration into the PTEN gene, coupled with clonal expansion of HIV-infected cells, may impair anti-tumor immune responses and promote cancer progression in PWH.

## Simple Summary

This study reveals that HIV integration into the Phosphatase and Tensin Homolog (PTEN) gene in immune cells can disrupt PTEN expression, potentially impairing the immune response against cancer and promoting cancer progression in people with HIV (PWH). This finding highlights a novel mechanism by which HIV may influence cancer development, providing new insights that could inform future cancer immunotherapy and management strategies for PWH.

## 1. Introduction

The health of people with HIV (PWH) has significantly improved with the advent of combination antiretroviral therapy (ART) [1], but PWH exhibit increased mortality, necessitating a focus on the prevention, diagnosis, and treatment of non-AIDS-related causes of death. Lung cancer is a particularly noteworthy cause of this increased mortality, as it is the third most common cancer in PWH, the leading non-AIDS-related cancer in PWH, and the second largest cause of cancer-related deaths in PWH [2]. Specifically, the incidence of lung cancer in PWH is elevated 1.5- to 3-fold compared to the general population, and while smoking is more prevalent among PWH, HIV itself is still an independent risk factor for lung cancer [3,4].

A crucial aspect of HIV persistence is the integration of HIV DNA into the genome of susceptible immune cells, such as CD4+ T cells and macrophages. HIV most commonly integrates into regularly expressed genes, including “oncogenes” [5,6]. Once integrated, these cells often undergo clonal expansion that is driven by antigen-specific and bystander inflammation [7]. Further, viral integration into genes regulating cell proliferation, survival, and immune responses [8,9,10,11,12,13,14], may confer a survival advantage to HIV-infected cells.

Within the tumor microenvironment (TME), a network of immune cells, stromal cells, and extracellular matrix components interact with tumor cells, fostering tumor growth and metastasis [15]. In PWH, the presence of clonally expanded HIV-infected cells within the TME likely complicates this interaction, as these HIV-infected cells may disrupt normal immune cell function, perpetuate chronic inflammation, and evade immune surveillance [16,17]. Such activities likely facilitate tumor cell survival and proliferation [18].

Tumorigenesis is driven by genetic and epigenetic processes involving oncogene activation and tumor suppressor gene inactivation [19]. Among the key tumor suppressors, Phosphatase and Tensin Homolog (PTEN) is frequently inactivated in human cancers. Even a partial loss of PTEN function can lead to neoplastic transformation [20,21]. PTEN expression in cancer cells attracts different immune cell populations to the TME, while its function in immune cells regulates their activation status, contributing to an immunosuppressive TME [22,23]. PTEN also regulates angiogenesis [24] and modulates the TME through the production of specific soluble factors (e.g., IL8, PD-L1) that alter stromal and immune cell infiltration. Finally, altered PTEN expression is associated with poor prognosis in lung cancer [12,25], frequently characterized by aberrant AKT activation due to loss of PTEN or PIK3CA/AKT1 activating mutations, often leading to treatment resistance [26].

In this report, we characterize a male PWH with high-grade neuroendocrine small cell lung carcinoma by mapping the landscape of HIV integration and the clonal expansion of HIV-infected cells in the TME, adjacent non-TME lung tissue, blood, and other peripheral tissues. Our analysis identified strong associations between evidence of HIV integration (i.e., HIV p24 expression) and reduction in PTEN expression, suggesting a potential impact on cancer progression.

## 2. Materials and Methods

### 2.1. Sample Collection and Processing

Peripheral blood mononuclear cells (PBMC) and tissue samples were obtained from one PWH enrolled in the Last Gift program [27]. The participant was closely monitored during the final phase of life, providing: (i) comprehensive clinical and socio-demographic data, including information about ART usage, cancer treatments, surgical procedures, and co-infections; (ii) blood samples prior to death; and (iii) consent for whole-body donation upon death for research purposes. The Last Gift rapid research autopsy protocol aims to collect high-quality tissue samples within six hours of death to minimize post-mortem degradation [28]. Immediately following death, the body was swiftly transported to the morgue, where a full autopsy was performed to obtain formalin-fixed paraffin-embedded tissue for histological analysis and snap-frozen tissue samples preserved in liquid nitrogen.

### 2.2. DNA Extraction, Purification and Quantification

Genomic DNA was isolated from 5 million PBMCs and snap-frozen tissue samples using the QIAamp DNA Mini Kit (Qiagen, Hilden, Germany, cat#51306), following the manufacturer’s instructions. DNA was then concentrated via precipitation. DNA concentrations were measured using the NanoDrop One spectrophotometer (ThermoScientific, Waltham, MA, USA). Droplet digital PCR (ddPCR) was employed to quantify HIV DNA levels in the extracted genomic DNA, using the BIO-RAD QX200 Droplet Reader (Bio-Rad Laboratories, Inc. Hercules, CA, USA) [29]. Copy numbers were calculated as the average of three replicate PCR assays and normalized to one million cells, as estimated by RPP30 quantification (total cell count control) [29,30].

### 2.3. HIV Integration Site (IS) Sequencing and Analyses

HIV IS sequencing was performed using an adapted version of the protocol developed by Wells et al. [31]. In brief, HIV IS were amplified using a modified linker-mediated PCR protocol, which enhances specificity and sensitivity. This method selectively amplifies host-provirus junctions at both the 3′ and 5′ long terminal repeats (LTRs), thereby increasing the likelihood of detecting valid integration events. We designed participant specific primers for both the 3′ (CACTTTTAGTCAGTGTGGAAAATC) and the 5′ (TCTTGTCTTTGCTGGGAGTGAATTA) LTRs. Genomic DNA was extracted from HIV-infected cells, and sequencing libraries were prepared using the NEBNext Ultra II FS DNA Library Prep Kit (New England Biolabs (NEB), Ipswich, MA, USA) designed for Illumina platforms. The number of integrated HIV proviruses in the samples was precisely quantified using a digital droplet PCR (ddPCR) approach. The Illumina sequencing data were analyzed using a custom pipeline adapted from Wells et al. [31]. The pipeline consists of several major steps: (1) Sample demultiplexing. (2) Pre-alignment trimming and filtering. (3) Genome alignment. (4) Post-alignment filtering and removal of artifacts. (5) Report generation. The analytical pipeline was implemented in Perl and utilizes BLAT [32], a fast alignment tool similar to BLAST (2.15.0), to align sequencing reads to the reference genome. Due to sonication, DNA is randomly fragmented, resulting in variable fragment lengths at identical integration sites. These unique fragment lengths serve as an indicator of the number of infected cells harboring a given integration site, a measure referred to as sonic abundance. Sonic abundance was estimated by counting the distinct fragment lengths associated with each integration site, thus inferring the number of infected cells at that site [33]. The distribution of the HIV IS along the human genome was plotted using the circos genomic R package (1.2.2) [34]. HIV IS were annotated using the ChIPseeker (3.21) peak annotation R package [35].

### 2.4. Statistical Methods

To compare the proportion of clonally expanded cells in TME versus non-TME tissues (lungs, lymph nodes and parietal cortex), we applied a paired *t*-test. Association between reservoir size and clonality was evaluated with a generalized mixed model. All analyses were performed in R.

### 2.5. Tissue Preparation

Snap-frozen tissues in liquid nitrogen were transferred to a screw-capped cryo-vial chilled on dry ice and warmed to −20 °C overnight in a freezer. Tissue samples were placed on ice and fixed in 4 °C (ice-cold) 10% Buffered formalin. Tubes were filled completely with fixative, maintaining at least 10:1 fixative/tissue volume ratio. Samples were stored at 4 °C prior to processing. Fixed tissue was washed with PBS twice for 5 min and placed in increasing sucrose concentrations (10%, 20%, and 30% sucrose diluted in PBS) rocking at room temperature for at least one hour or until tissue sunk in solution. Tissue was removed and submerged in a tissue mold (22 × 22 × 20 mm embedding mold, Fisher Scientific (Waltham, MA, USA)) filled with optimal cutting temperature (OCT) compound (Tissue Plus, Fisher HealthCare (Waltham, MA, USA)) and frozen at −80 °C. 10 μm tissue sections were cut with a Leica CM 1950 cryostat and mounted on Superfrost Plus slides (Fisher Scientific), air dried and stored at −80 °C until staining.

### 2.6. Tissue Immunostaining

Tissue slides were defrosted and incubated at 37 °C for 30 min prior to washing three times with PBS for five minutes each. Tissue was permeabilized with 0.25% Triton-X in PBS for 30 min at room temperature, then blocked at room temperature in 0.1% Triton-X, 4% FBS, 0.01% Sodium Azide, 1:100 anti-human FcR (Miltenyi Biotec, Bergisch Gladbach, Germany) in PBS for 30 min. Tissues were stained with primary antibodies: human anti-p24 (1:1000, MediMabs, Montreal, QC, Canada), rabbit anti-human PTEN (1:100, Biolegend, San Diego, CA, USA), and mouse anti-human EGFR (1:50 conjugated with AlexaFlour 647, Biolegend) diluted in blocking buffer, and incubated overnight at 4 °C protected from light. Tissues were washed three times for 5 min in PBS and incubated with secondary antibodies (anti-rabbit AlexaFlour 568 and anti-human AlexaFlour 488 diluted 1:1000 in PBS, with 1:100 anti-human FcR and 4% FBS) for one to two hours at room temperature protected from light. Tissues were washed three times with PBS, treated with Vector TrueVIEW Autofluorescence Quenching kit (Vector Laboratories, Newark, CA, USA) and counterstained with DAPI for 5 min. Tissues were mounted in prolong Gold Antifade reagent (Life Technologies, Carlsbad, CA, USA) under a coverslip and sealed with nail polish.

### 2.7. Confocal Microscopy

Immunostained slides were imaged with a Zeiss LSM 880 or LSM 900 confocal microscope with a 20× air objective. Tiled images were acquired to capture maximal area of immunostained tissue sections. Tiled images were fused using Zeiss Zen Blue software (3.6).

### 2.8. Image Analysis

Stitched confocal images were analyzed in Imaris software. Tumor boundaries were segmented using the volumetric surface tool and manual thresholding of DAPI fluorescence. Fluorescence of PTEN and EGFR was quantified using the spots tool in Imaris using an estimated sphere diameter of 3 μm. Mean fluorescence intensity (MFI) within each sphere was recorded relative to the position within or outside the tumor boundaries and percent expression was graphed in Graphpad Prism 10.

### 2.9. Resource Availability

Anonymized sequencing data was deposited at SRA and will be publicly available at the date of publication. Accession numbers are listed in the key resources table (Table S1).

## 3. Results

### 3.1. Participant Characteristics and Medical History

The study participant was a 74-year-old white non-Hispanic male diagnosed with HIV in 2000. Comorbidities included hypertension, hyperlipidemia, and coronary artery disease. He was never a smoker or diagnosed with alcohol use disorder. He started on ART in 2000 (nadir CD4 300 cells/μL) and remained virally suppressed until the time of death with no history of opportunistic infections. In July 2021, he was diagnosed with a high grade neuroendocrine small cell lung carcinoma with extension in lymph nodes and brain. Molecular profiling identified an epidermal growth factor receptor (EGFR) L858R mutation, a single nucleotide variant in exon 21 in the tyrosine kinase domain of the EGFR.

Following his cancer diagnosis, he underwent 4 cycles of chemotherapy with concurrent radiation from August 2021 to December 2021, followed by 2 doses of Lurbinectedin in June and July 2022. In 2021, He voluntarily enrolled in the Last Gift program, an end-of-life translational research program which aims to understand HIV dynamics throughout the human body [28,36,37,38].

He died in October 2022. Two days prior to the autopsy, blood was collected. During the rapid autopsy, which allowed specimens to be collected within six hours of death, paired tumoral and adjacent non-tumoral tissues samples were collected from lung, lymph nodes and brain. These samples were analyzed for HIV integration and identification of clonally expanded HIV-infected immune cells. Paired lung tumor and adjacent healthy lung underwent IF to further assess PTEN, EGFR and HIV p24 gene expression.

### 3.2. Landscape of HIV Integration and Clonal Expansion of HIV Infected Cells

Analyses of HIV IS sequencing data identified 174 unique IS (313 total, see Figure S1). Most proviral integrations were in introns, with a mean of 55.3% [SD 10.4%, IQR 13.5%], varying from 38% in the parietal cortex (TME) to 69.8% in the lung (non-TME). See Figure S2 for genomic features associated with HIV IS.

**Figure 1:**
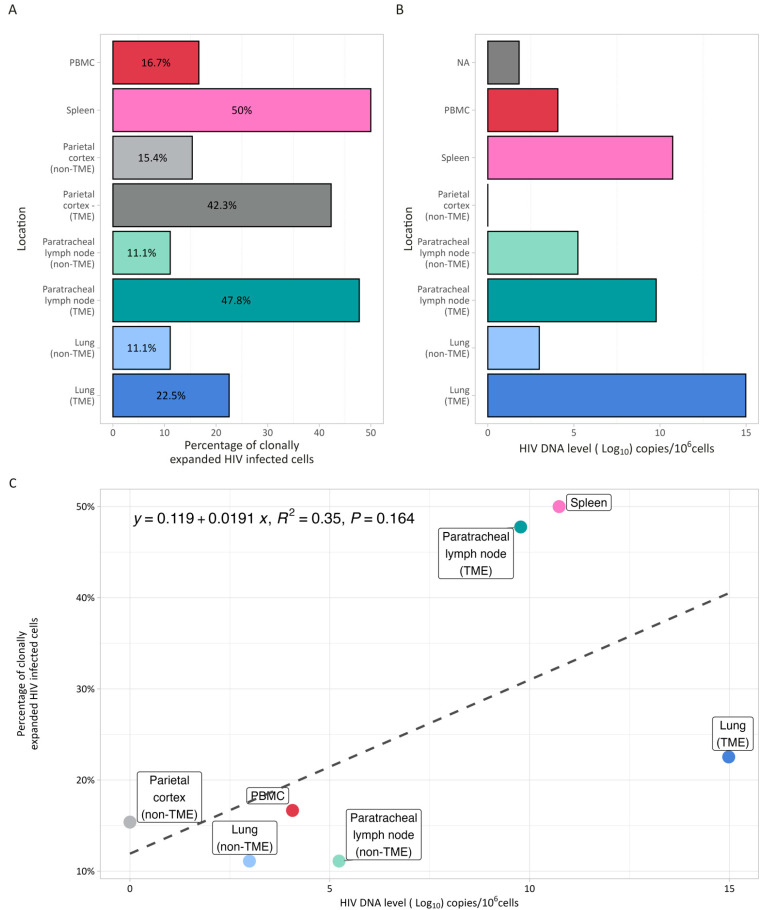
HIV reservoir size and clonality. (**A**) Percentage of clonally expanded cells identified by integration site sequencing in blood, spleen, paired TME and non-TME parietal cortex, lymph nodes and lung. (**B**) HIV DNA level (expressed in log_10_ copies/10^6^ cells) determined by digital droplet PCR in the same samples. Compared to non-TME tissues, paired TME tissues from the lung, lymph nodes, and parietal cortex had a higher proportion of clonally expanded HIV-infected cells and higher HIV DNA levels, although these differences were not statistically significant (*p* = 0.07 and *p* = 0.18). (**C**). Association between HIV DNA level (x axis) and percentage of clonally expanded cells (y axis). There was no significant association between HIV reservoir size (DNA level) and the percentage of clonally expanded HIV-infected cells.

Since most of the latent HIV reservoir is maintained through the clonal expansion of HIV-infected immune cells, and HIV integration has been linked to the improved survival of infected cells [39], we identified the unique HIV IS in clonally expanded HIV-infected cells in each sample. Overall, 29.9% (52/174) of HIV integrations were found in clonally expanded cells (Figure 1A). When comparing adjacent tumor and non-tumor tissues from the lung, lymph nodes, and parietal cortex, there was a higher proportion of clonally expanded HIV-infected cells and higher HIV DNA levels in tumor tissues versus non-tumor tissues, although these differences were not statistically significant (*p* = 0.07 and *p* = 0.18), and there was no significant association between HIV DNA levels and clonality (Figure 1B,C).

**Figure 2:**
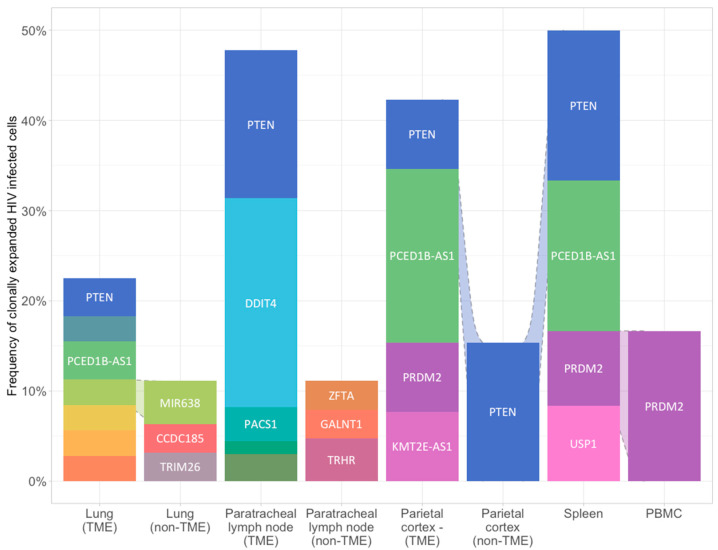
Proviral HIV integration in HIV infected clonally expanded cells across blood and paired tumoral and non-tumoral tissues. Relative sonic abundance for each HIV integration site (IS) is shown in clonally expanded cells. Clones consisting of 5% or more of the total relative sonic abundance are labeled with the integrated gene. Different colors indicate different integration sites. See method for details.

The most common gene with HIV integration in clonally expanded cells across samples was PTEN. This integration occurred in the intronic region at position chr10-87,869,734. Viral integration was found in cells of the tumor regions of lung, paratracheal lymph node, and both tumor and non-tumor regions of parietal cortex and spleen. Integration was not identified in non-tumor tissues of lung or lymph node, or in PBMCs. Clonally expanded HIV-infected cells with integration into PTEN accounted for 4.2% to 16.7% of all HIV-infected cells across all samples (Figure 2).

### 3.3. Tumor Heterogeneity and Gene Expression

**Figure 3:**
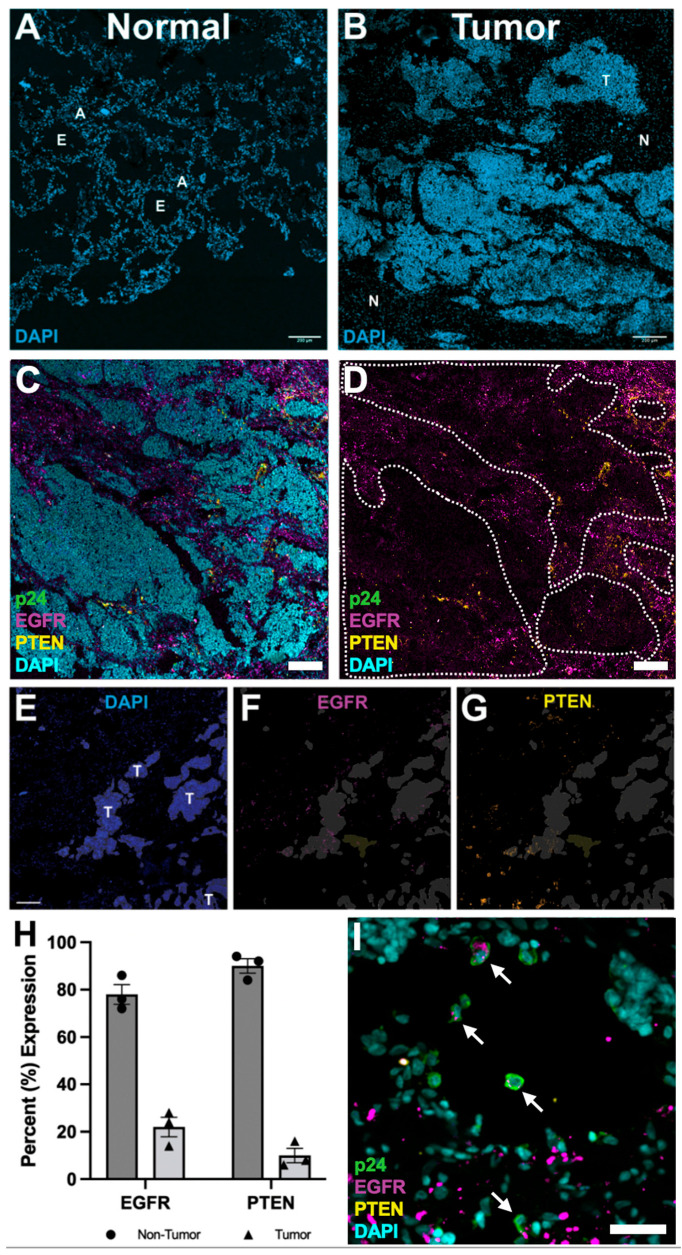
Immunofluorescence analysis of tumor tissue. (**A**,**B**) Tissue architecture of normal and tumor tissue from PWH nuclear stained with DAPI to show cell organization. Normal tissue shows defined tissue architecture revealing epithelial airway (**E**) and alveolar (**A**) regions. Tumor regions (T) show loss of regular tissue architecture, revealing dense aggregations of nuclei compared to normal regions (N). Scale bar = 200 μm. (**C**) Tumor region stained for nuclei (DAPI/cyan), HIV p24 (green), EGFR (magenta), and PTEN (yellow). Scale bar = 200 μm. (**D**) Same image as (**C**) with DAPI staining removed. Tumor regions were outlined (white dotted line). (**E**–**G**) Automated segmentation of tumor regions (**E**) to analyze EGFR (**F**) and PTEN (**G**) expression associated with tumor regions (T). Scale bar = 100 μm. (**H**) Percent expression of EGFR and PTEN within non-tumor (black circles/dark gray bars) and tumor (black triangles/light gray bars) regions. Error bars = SEM. (**I**) Immunofluorescence of HIV p24 (green), nuclei (DAPI/cyan), PTEN (yellow), and EGFR (magenta). White arrows indicate HIV p24 expressing cells co-expressing EGFR. Scale bar = 50 μm.

We next investigated the tissue expression patterns of PTEN, identified as a major site of HIV integration, and EFGR, whose mutation was identified prior to death in this participant. Although EGFR mutations are not typically associated with neuroendocrine small cell lung carcinoma and no HIV integration was detected in the EGFR locus, the presence of a deleterious EGFR mutation may contribute to reduced EGFR expression in tumor regions. To interrogate the spatial landscape of PTEN, EGFR, and HIV expression in tumor tissue compared to non-tumor tissue, flash frozen tissues sections were prepared for IF. Confocal microscopy of tissue sections stained for nuclei revealed visually distinct tissue architecture in non-tumor lung regions compared to tumor regions (Figure 3A,B). Neoplastic regions showed dense aggregations of cells with loss of distinct airway epithelium and alveolar architecture compared to non-tumor regions.

Initial IF surveys of solid lung tumor regions showed little HIV replication, as indicated by the lack of HIV p24 expression, and reduced EGFR expression compared to non-tumor regions (Figure 3C,D). Interestingly, PTEN expression was reduced in tumor regions compared to non-tumor regions. To validate these visual observations, EGFR and PTEN expression levels in tumor and non-tumor regions were quantified using semi-automated segmentation to distinguish spatially heterogenous expression patterns in an unbiased fashion (Figure 3E–G). These analyses revealed reduced expression of PTEN and EGFR in tumor regions compared to non-tumor regions (Figure 3H).

Although ART effectively suppresses HIV replication, transcriptionally active provirus can persist, as evidenced by the detection of rare HIV p24-positive cells within and adjacent to tumor regions (Figure 3I) [40,41]. These HIV p24-positive cells rarely expressed PTEN but frequently expressed EGFR. While the small number of HIV p24-positive cells limits definitive interpretation, these findings suggest that HIV integration into the PTEN locus could contribute to its reduced expression within the tumor microenvironment.

## 4. Discussion

This study explored the landscape of HIV integration and clonal expansion in a PWH with high-grade neuroendocrine small cell lung carcinoma and found 174 unique HIV IS in HIV-susceptible cells. There was a high frequency of these integrations within the PTEN gene in clonally expanded HIV-infected cells, which were found predominantly in tumor tissues. Further IF and confocal microscopy confirmed a heterogeneous loss of PTEN expression in tumor regions compared to non-tumor regions, and this loss of PTEN expression was associated with HIV p24 expression, suggesting that HIV integration into PTEN may impact its expression.

These findings demonstrate a clear differential impact of HIV infection on the immune microenvironment and highlight a potential mechanism by which HIV may contribute to cancer progression in PWH. The observed large clonal expansions of HIV-infected T cells within the TME (Figure 1A) suggest that infiltrating HIV-infected T cells can alter the immune composition of the TME. Such alterations may compromise the immune system’s ability to mount effective responses against novel antigens, including tumor-associated antigens, thereby impairing immune surveillance and hindering the elimination of newly transformed cancer cells. Moreover, the persistent state of chronic inflammation driven by ongoing HIV replication can paradoxically lead to immunosuppression [42]. This immunosuppressive environment facilitates the unchecked progression of the tumorigenic process, enabling transformed cells to evade immune control and proliferate unimpeded [43,44,45].

PTEN, a well-known tumor suppressor gene, is essential for regulating both intrinsic cancer cell signaling and the immune landscape within tumors [46,47]. Its loss has far-reaching consequences for immune cell function, the TME, and immunotherapy responses, particularly in lung cancer. PTEN deficiency reduces CD8+ T cell activity, compromising the immune system’s ability to target tumor cells [48]. Simultaneously, PTEN loss promotes the infiltration of immunosuppressive myeloid-derived suppressor cells (MDSCs) and regulatory T cells (Tregs), while also altering cytokine production to favor an immunosuppressive milieu [49]. Beyond immune modulation, PTEN loss inhibits autophagy, which is crucial for T cell-mediated clearance of pathogens and cancer cells [50]. It also drives the release of exosomes carrying microRNAs that further suppress PTEN in MDSCs, enhancing immune evasion [50]. Collectively, these effects create an immunosuppressive TME that has been associated with resistance to immune checkpoint blockade therapies, such as anti-PD-1 treatment [51,52].

In lung cancer, PTEN loss poses a major challenge to effective antitumor immunity, undermining both immune surveillance and immunotherapy outcomes [48,51]. Targeting PTEN loss holds promise as a therapeutic strategy: a recent study by Zhang et al. using an in situ lung cancer model demonstrated that precise delivery of PTEN mRNA restored PTEN function, alleviated immunosuppression, and improved the local immune environment in the lungs [52]. Hence, by integrating into regulatory genes such as PTEN, HIV could disrupt cellular pathways that promote neoplastic transformation and support tumor maintenance. This is particularly significant, as PTEN loss has been implicated in the development of numerous cancers and is known to drive resistance to therapy via AKT and other signaling pathways [48,53]. Such disruptions may also confer resistance to ionizing radiation, potentially diminishing the efficacy of radio-sensitizing agents like KU-60019 [12]. These findings have important implications for the treatment of cancer in PWH.

While this study can only evaluate associations rather than establish causation, its findings suggest a potential mechanism by which HIV may influence cancer progression through disruption of key regulatory genes in immune cells—particularly those within the TME. This possibility is supported by the heterogeneity in PTEN and EGFR expression observed between tumor and non-tumor tissues in this case. Specifically, the reduced PTEN expression in tumor regions, combined with the presence of p24-positive cells, underscores the persistent impact of HIV on cellular dynamics within the TME. These observations provide further insight into mechanisms potentially driving tumor progression in PWH and highlight opportunities for therapeutic strategies aimed at restoring PTEN function, ranging from genetic interventions to precision medicine approaches tailored to PTEN functional status [54].

Moreover, while our findings in this single case report are limited to lung cancer, the observed association between HIV integration within PTEN and its downregulation raises the possibility that similar mechanisms may be involved in other cancer types impacting PWH. The loss of PTEN function can result from diverse mechanisms, including point mutations [55], promoter methylation [56], microsatellite instability [57], and miRNA upregulation [58]. PTEN loss was previously described in lung cancer and several additional tumor types, most notably in glioblastoma [59,60], endometrial [61], ovarian [62] and prostate cancers [63], and to a lesser extent in breast and colon cancers [64,65]. Future studies investigating PTEN expression across different cancer types in larger cohorts of PWH will be critical to delineate the broader relevance of these observations and to determine whether PTEN downregulation consistently associates with specific cancer types in this population. Our findings also resonate with earlier studies demonstrating that viral integration events can drive clonal expansion of infected cells [66] and that HIV-infected immune cells can infiltrate tumors and alter the TME [67].

While our observations are limited to a single PWH, they suggest a provocative mechanism by which HIV may affect the TME. If this mechanism indeed contributes to cancer progression in PWH, the identification of clonally expanded, HIV-infected cells with specific gene integrations—such as PTEN—could serve as a biomarker for assessing cancer risk and tailoring screening and treatment strategies for PWH, who carry a higher cancer burden compared to people without HIV.

## 5. Conclusions

Defining the landscape of HIV integration within key regulatory genes, such as PTEN, provides important insights into how HIV may influence the tumor microenvironment and contribute to cancer progression in PWH. Although more research is needed to confirm these associations and understand their functional implications, our findings lay the groundwork for future studies with larger cohorts of PWH. Such studies will be critical for validating these observations, assessing their generalizability, and evaluating the clinical utility of screening for HIV integration sites as cancer susceptibility biomarkers. Ultimately, these efforts could lead to valuable tools for cancer risk assessment and management in PWH, advancing both our understanding of HIV-related cancer pathogenesis and the development of novel therapeutic strategies that target the complex interplay between HIV persistence and cancer progression.

## Data Availability

Anonymized sequencing data have been deposited at SRA and will be publicly available as of the date of publication. Accession numbers are listed in the key resources table (Table S1).

## Supplementary Materials

The following supporting information can be downloaded at: https://www.mdpi.com/article/10.3390/curroncol32070389/s1, Figure S1: HIV integration site distribution across chromosomes in blood, spleen and tumor and non-tumor tissues; Figure S2: Feature distributions; Table S1: Key resources table.

## Abbreviations

The following abbreviations are used in this manuscript:

PWH	People with HIV
ART	Antiretroviral Therapy
TME	Tumor Microenvironment
PTEN	Phosphatase and Tensin Homolog
PD-1	Programmed Cell Death Protein 1
IF	Immunofluorescence
EGFR	Epidermal Growth Factor Receptor
PBMC	Peripheral Blood Mononuclear Cells
IS	Integration Site
HIV DNA	Human Immunodeficiency Virus Deoxyribonucleic Acid
p24	HIV p24 Capsid Protein
IL8	Interleukin 8
PD-L1	Programmed Death-Ligand 1
PIK3CA	Phosphatidylinositol-4,5-Bisphosphate 3-Kinase Catalytic Subunit Alpha
AKT1	AKT Serine/Threonine Kinase 1
SRA	Sequence Read Archive
UCSD	University of California San Diego
ddPCR	Droplet Digital Polymerase Chain Reaction
LTR	Long Terminal Repeat
OCT	Optimal Cutting Temperature (compound)
PBS	Phosphate-Buffered Saline
FBS	Fetal Bovine Serum
DAPI	4′,6-Diamidino-2-Phenylindole
MFI	Mean Fluorescence Intensity
NIH	National Institutes of Health
NIDA	National Institute on Drug Abuse
CFAR	Center for AIDS Research
