# Supplementary material for: HIV Integration into the PTEN Gene and Its Tumor Microenvironment Implications for Lung Cancer
Source: Curr Oncol. 2025 Jul 4;32(7):389. doi: 10.3390/curroncol32070389 (PMC12294024; doi:10.3390/curroncol32070389)
Supplement: Supplementary file 1 [file curroncol-32-00389-s001.zip › curroncol-3667743-supplementary.pdf]

Supplementary Materials

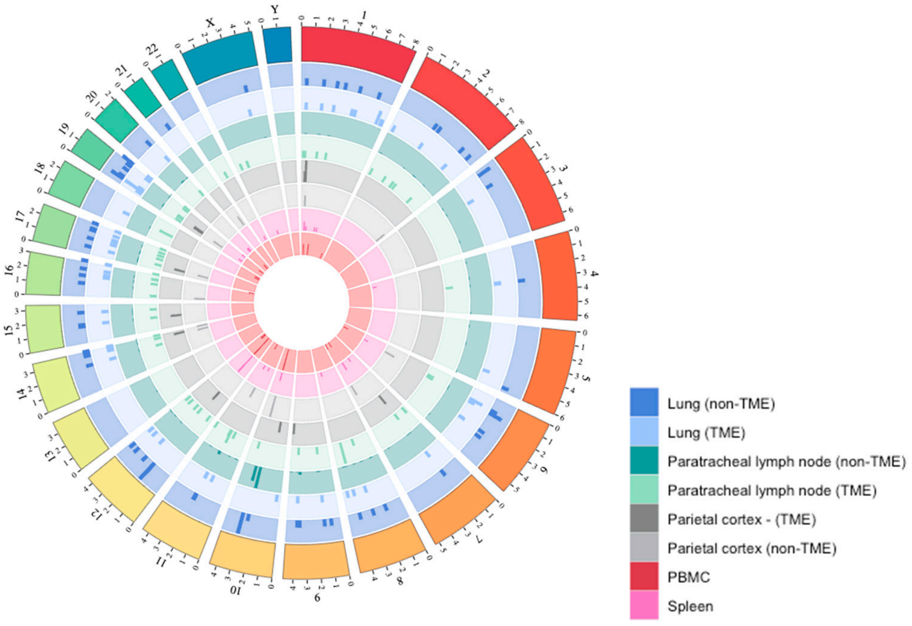

Figure S1. HIV integration site distribution across chromosomes in blood, spleen and tumor and non-tumor tissues.

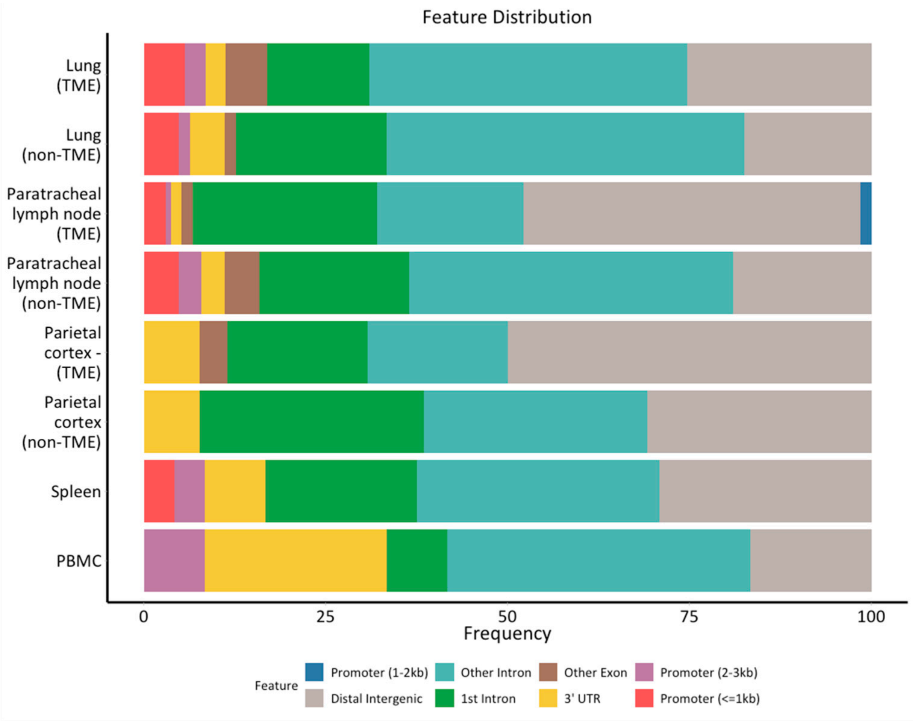

Figure S2. Feature distributions. The percentage of total integration sites combined in blood, spleen and tumor and non-tumor tissues is displayed for each feature annotation analysis was performed using the ChIPseeker peak annotation R package [1].

Table S1. Key resources table.

REAGENT or RESOURCE	SOURCE	IDENTIFIER *
PBMC	PBMC	PRJNAxxxx
LNGT	Lung (tumor)	PRJNAxxxx
LNG	Lung	PRJNAxxxx
LNT	Lymph nodes (tumor)	PRJNAxxxx
LN	Lymph nodes	PRJNAxxxx
PCTT	Parietal cortex (tumor)	PRJNAxxxx
PCT	Parietal cortex	PRJNAxxxx
SPL	Spleen	PRJNAxxxx

* Identifiers will be made available at the time of publication.

Reference

1. Yu, G.; Wang, L. G.; He, Q. Y., ChIPseeker: An R/Bioconductor package for ChIP peak annotation, comparison and visualization. *Bioinformatics* **2015**, *31*, 2382-3.
